# Evaluation of corneal tomography in children and adolescents without ocular or systemic allergy

**DOI:** 10.5935/0004-2749.2021-0048

**Published:** 2022-07-04

**Authors:** Roddie Moraes Neto, Gabriel Bordignon, Nayara Teixeira Flügel, Vinícius Tadashi Okuyama, Cristine Secco Rosário, Crislaine Caroline Serpe, Nelson Augusto Rosário Filho, Herberto José Chong Neto, Glauco Henrique Reggiani Mello

**Affiliations:** 1 Faculdade de Medicina, Universidade Federal do Paraná, Curitiba, PR, Brazil; 2 Department of Ophthalmology-Otorhinolaryngology, Health Sciences Sector, Universidade Federal do Paraná, Curitiba, PR, Brazil; 3 Department of Allergy and Immunology, Health Sciences Sector, Universidade Federal do Paraná, Curitiba, PR, Brazil

**Keywords:** Cornea, Corneal topography, Astigmatism, Conjunctivitis, allergic, Tomography, Keratoconus/diagnosis, Humans, Child, Adolescent, Córnea, Topografia corneana, Astigmatismo, Con­juntivite alérgica, Tomografia, Ceratocone/diagnóstico, Humanos, Criança, Adolescente

## Abstract

**Purpose:**

To determine normal corneal tomographic parameters in children and adolescents without corneal disease or atopy diagnosis.

**Methods:**

This descriptive cross-sectional study evaluated patients aged 8-16 years who underwent a complete slit-lamp biomicroscopic examination and tomographic corneal evaluation by a dual Scheimpflug analyzer, excluding those with ocular disease (including allergic conjunctivitis) or a positive prick test for systemic atopies.

**Results:**

A total of 170 patients were evaluated, and 34 patients (68 eyes) were analyzed once the exclusion criteria were applied. The sample’s mean age was 10.76 ± 2.31 years; with 19 (55.9%) men and 15 (44.1%) women. The mean keratometry in the flat meridian (K_flat_), steep meridian (K_steep_), and maximum (K_max_) were 42.37 ± 1.63D, 43.53 ± 1.65D, and 43.90 ± 1.73D, respectively. The mean values for corneal asphericity (ε^2^) and thinnest point were 0.28 ± 0.11 and 550.20 ± 37.90 µm, respectively. The inferior-superior asymmetry ratio (I-S) and coma were 0.74 ±0.59D and 0.28 ± 0.12D, respectively.

**Conclusion:**

The knowledge of normal corneal tomographic parameters and their variation in children and adolescents without corneal disease or atopy may be useful for diagnosing keratoconus and initiating early disease treatment.

## Abstract (pt)

**Objetivo:**

Identificar parâmetros tomográficos de normalidade em córneas de crianças e adolescentes sem a presença de atopias sistêmicas e alergias oculares.

**Métodos:**

Este estudo descritivo transversal avaliou pacientes com idade entre 8 e 16 anos que foram submetidos a exame biomicroscópico completo por lâmpada de fenda e avaliação tomográfica da córnea por tomógrafo dual Scheimpflug, excluindo pacientes com doença ocular (incluindo conjuntivite alérgica) ou *prick test* positivo para atopias sistêmicas.

**Resultados:**

Cento e setenta pacientes foram avaliados e após cumpridos os critérios de exclusão, 34 (68 olhos) foram analisados. A média etária da amostra foi 10,76 ± 2,31 anos; 19 (55,9%) eram meninos e 15 (44,1%) meninas. A média da ceratometria em dioptrias (D) no meridiano mais plano (K_flat_), mais curvo (K_steep_) e máxima (K_max_) foram 42,37 ± 1,63D, 43,53 ± 1,65D e 43,90 ± 1,73D, respectivamente. Os valores médios da asfericidade corneana (ε^2^) e do ponto mais fino da córnea foram 0,28 ± 0,11 e 550,20 ± 37,90 micras (µm). A assimetria corneana inferior-superior (I-S) e coma foi em média 0,74 ± 0,59D e 0,28 ± 0,12D, respectivamente.

**Conclusão:**

O conhecimento dos valores médios e sua variação de parâmetros tomográficos da córnea em crianças e adolescentes sem atopias sistêmicas ou alergias oculares pode ser útil para o diagnóstico precoce do ceratocone e o seu tratamento em estágio inicial.

## INTRODUCTION

Ectasias are corneal changes induced by structural weakening. Keratoconus, the most prevalent ectasia, is a bilateral, progressive, and asymmetric disorder asso­ciated with structural changes in the organization of corneal collagen. Classically, the disease manifests itself in the second decade of life, when the cornea assumes a more conical form, resulting in irregular astigmatism, progressive myopia, thinning of the cornea, and a concomitant decline in visual acuity^(1)^.

Pediatric keratoconus is more aggressive than adult keratoconus because the young cornea is biomechanically less resistant, resulting in rapid progression of ectasia and a seven-fold increase in the need for corneal transplantation^(2)^. In addition, the frequent coexistence of ocular pathologies, such as atopy and vernal keratoconjunctivitis, has been associated with faster progression and long-term keratoconus complications in children^(1,3)^.

The study of corneal regularity can be carried out using Placido, Scheimpflug, or Optical coherence tomo­graphy images^(4-6)^. The combination of Placido and Scheimpflug enables the detection of subtle changes in corneal conformation, regularity, and thickness, which is important for early diagnosis of keratoconus and other corneal ectasias^(5-7)^.

Therefore, the knowledge of corneal topographic and tomographic parameters considered normal in children and adolescents without corneal disease or systemic atopy may be crucial in identifying subclinical changes in the cornea and helping in the early diagnosis and treatment of ectasias in young patients.

## METHODS

A descriptive cross-sectional study was conducted at the *Hospital Universitário do Complexo Hospitalar das Clínicas da Universidade Federal do Paraná* (CHC-UFPR), Curitiba-PR-Brazil, from November 2018 to April 2019. This research was approved by the Research Ethics Committee of CHC-UFPR, under protocol no. 2855765. The ethical principles of privacy and confidentiality of the collected data were respected. All participants were minors and signed the informed assent form (TALE) and their respective parents signed the Informed Consent Form.

The inclusion criteria were patients aged 8-16-year-old referred to the ophthalmology and immunology outpatient clinics of CHC-UFPR between November 2018 to April 2019 and presented a standardized and reliable tomographic corneal examination, as recommended by the manufacturer. Exclusion criteria were: patients with allergy (positive prick test or allergy identified through the application of a previously validated questionnaire)^(8)^, history of ocular surgery or corneal disease, patients with keratoconus diagnosed by tomography, presence of incomplete data or images with low-reliability index.

Two ophthalmologists from the CHC-UFPR performed a complete ophthalmologic examination in both eyes, which included slit-lamp biomicroscopy and corneal tomography using the Galilei G6 system (Zeimer Ophthalmic Systems AG, Port, Switzerland). Patients with any ophthalmologic abnormality who were referred to this research were directed to a specific ophthalmology specialist (GRM).

The tomographic data analyzed were as follows: a subjective evaluation by a single corneal specialist, keratometry in the flat and steep meridian and maximum keratometry (K_flat_, K_steep_, and K_max_, respectively), asphericity (ε^2^), Corneal Thinnest Point (CTP), Keratoconus Probability Index (KPI), inferior-superior asymmetry ratio (I-S), Best-Fit Toric Aspheric at its maximum posterior elevation (BFTA/ MPE), Asphericity Asymmetry Index (AAI) or Kranemann-Arce Index, Cone Location and Magnitude Index (CLMI.x), best-fit sphere at its maximum posterior elevation (BFS MPE), the radius of the best-fit sphere (BFS MPE radius), and coma.

A single allergist specialist from the CHC-UFPR performed the allergic skin prick tests (SPT) - *ALK Sterile Disposable*, by FDA *Allergenic*^®^ IMMUNOTECH - and the allergic questionnaire. The questionnaire has already been validated and is based on three modules (asthma, rhinitis, and atopic eczema)^(9)^. Patients with more than three bouts of ocular itching in the previous year were judged to have allergic conjunctivitis^(8)^_._ The tests were applied following the manufacturer’s instructions. The standardized FDA *Allergenic^®^* aeroallergens used were: two species of mites (*Dermatophagoides pteronyssinus* 800 URC and *Blomia tropicalis* 800 URC), pollen mixture (grass pollen mix 400 µgP/mL), dog epithelium (*Canis familiaris* 400 µgP/mL), cat epithelium (*Felis domesticus* 400 µgP/mL), fungal mixture (*Aspergillus fumigatus, Alternaria alternata* 400 µgP/mL), and cockroaches (*Periplaneta americana*). The tested extracts were stored in FDA *Allergenic^®^* 2-mL vials and transported in a thermal container kept under refrigeration between 2 and 10°C.

To assess confidence level and relative error in the most important variables, a sample size determination for populational mean was undertaken. For K_max_, as an example, a sample size of 47 eyes was necessary to achieve 99% confidence level and 1.5% error, whereas, in corneal thinnest point, a sample size of 140 eyes was required to achieve the same confidence level and error. In addition, 51 eyes would give a 99% of confidence level and 2.5% of error in the latter.

Data were collected and organized in spreadsheets, processed, and compared using statistical software R (R Core Team, 2019)^(10)^, version 3.6.1. The Shapiro-Wilk test was used for statistical analysis of all the obtained data. For non-normally-distributed variables, the p-value was <0.05 and represented by median and interquartile rate (IQR). For normally distributed variables, the p-value was >0.05.

## RESULTS

A total of 170 patients were analyzed between November 2018 and April 2019. Of these, 12 were excluded because they presented low-reliability corneal tomographic indexes or incomplete data. Of the remaining 158 patients, 104 were then excluded due to the presence of allergic conjunctivitis and 20 due to systemic atopy.

K_max_, ε^2^, BFTA MPE, AAI, BFS MPE, and coma variables were normally distributed. K_flat_, K_steep_, CTP, KPI, I-S, and BFS radius were non-normally distributed and repre­sented by median and IQR.

**Figure 1:**
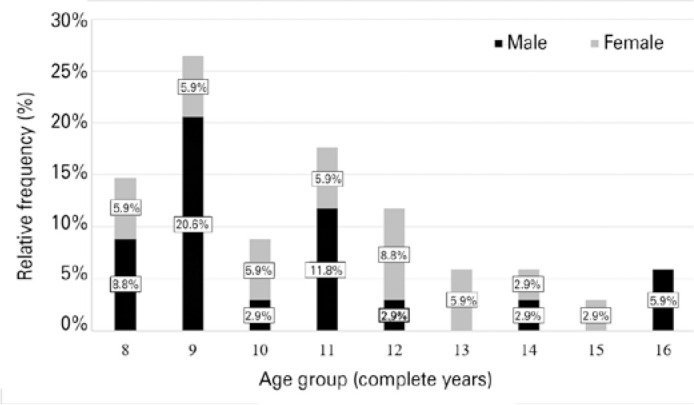
Relative frequency distribution of sample’s age and gender (n=34 patients).

The final sample included 68 eyes of 34 patients who did not present any abnormalities in the biomicroscopic examination and corneal tomography. Figure 1 presents the sample’s gender and age distribution. The mean age was 10.76 ± 2.31 years, ranging from 8 to 16 years, and male participants represented 55.9% of those evaluated.

Table 1 shows the tomographic parameters. For normally distributed variables, the median, mean ±standard deviation (SD), and range of variation are used, whereas, for non-normally distributed variables, the median and IQR are used. Regarding the corneal topographic pattern, it was observed that 16 (25%) of the 68 eyes analyzed had superior corneal asymmetry. Only one eye showed an abnormal CLMI index result, while another was considered borderline.

**Table 1:** Summary descriptive statistics of tomographic parameters

Index	Median	Mean ± SD	IQR	Range of variation	p-value
K_flat_ (D)	42.67		2.10	37.07 to 45.43	<0.05
K_steep_ (D)	43.97		2.30	39.17 to 46.52	<0.05
K_max_ (D)	44.25	43.90 ± 1.73	-	39.60 to 47.63	>0.05
Eccentricity (ε^2^)	0.28	0.28 ± 0.11	-	0.03 to 0.61	>0.05
Corneal thinnest point (µm)	559		40.75	462 to 612	<0.05
KPI (%)	0.35		6.28	0 to 31.30	<0.05
I-S (D)	0.64		0.71	0.04 to 2.66	<0.05
BFTA MPE (µm)	7	7.01 ± 2.55	-	2 to 12	>0.05
AAI (µm)	10	10.44 ± 5.05	-	0 to 22	>0.05
BFS radius (mm)	6.45		0.39	6.03 to 7.61	<0.05
BFS MPE (µm)	11	11.65 ± 4.17	-	4 to 23	>0.05
Coma (D)	0.26	0.28 ± 0.12	-	0.09 to 0.58	>0.05

IQR= interquartile range; K_flat_= Flat meridian keratometry; K_steep_= Steep meridian keratometry; K_max_= maximum keratometry; KPI= keratoconus probability index; I-S= inferior-superior asymmetry ratio; BFTA MPE= best-fit toric aspheric at its maximum posterior elevation; AAI= asphericity asymmetry index; BFS radius= best-fit sphere radius; BFS MPE= best-fit sphere at its maximum posterior elevation; D= diopter; µm: micrometers.

**Figure 2:**
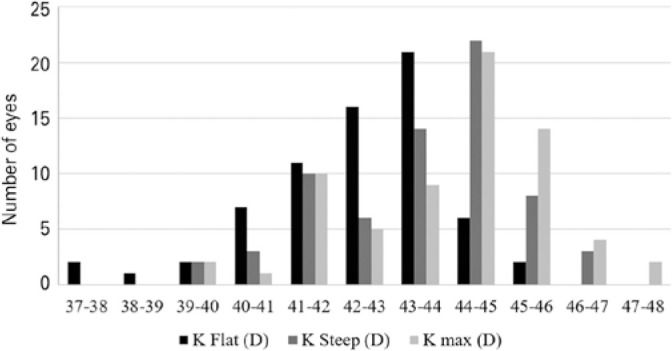
Keratometry frequency distribution (n=68 eyes).

Figure 2 summarizes the keratometry data of the 68 eyes analyzed. K_flat_, K_steep_ and K_max_ in diopters (D) were 42.37 ± 1.63 (range: 37.07-45.43), 43.53 ± 1.65 (range: 39.17-46.52), and 43.90 ± 1.73 (range: 39.60-47.63), respectively.

**Figure 3:**
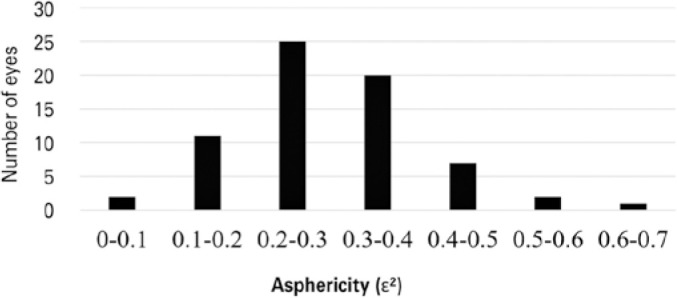
Asphericity frequency distribution (n=68 eyes).

The observed asphericity was 0.28 ± 0.11 (range: 0.03-0.61), with 66.2% of the evaluated eyes having values between 0.2 and 0.4 (Figure 3).

**Figure 4:**
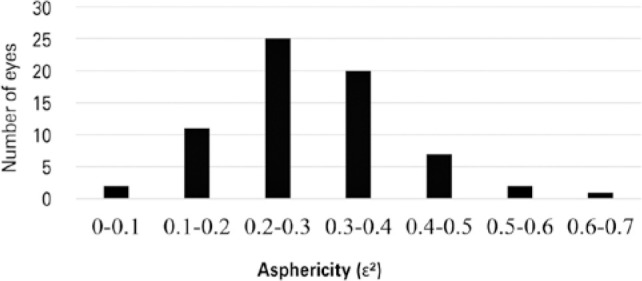
Corneal thinnest point frequency distribution (n=68 eyes).

The mean CTP was found to be 550.20 ± 37.90 µm in the analysis of corneal thinnest point (range: 462-612). It was observed that 64.7% of the patients were between 540 and 599 µm (Figure 4).

## DISCUSSION

Visual impairment in young individuals can have a severe influence on their social development, lowering their quality of life and subsequently their functional activity. Since pediatric corneas progress quickly to ectasia, delaying therapy owing to a difficult early keratoconus diagnosis may worsen the prognosis^(1)^.

For many years, penetrating keratoplasty was the sole therapeutic option for patients who experienced intolerance or low visual acuity with the use of contact lenses or glasses. However, in recent decades, several techniques, such as intrastromal corneal rings and crosslinking^(3)^, have been created and refined in an attempt to increase visual acuity and disease stability.

Patients deemed normal in the present study had an allergic skin prick test and a questionnaire that were both negative for the existence of atopy (allergic conjunctivitis, asthma, rhinitis, and atopic eczema). Since atopy was identified as one of the factors responsible for the chronic habit of eye rubbing, which is known as a risk factor for the development and progression of keratoconus, this patient selection was critical^(11)^.

Tomographic data, such as that supplied by dual Scheimpflug technology, greatly improve the screening of corneal ectasias. Tomography, in addition to the previous topography corneal analysis, provides information on the posterior cornea, elevation, and pachymetric distribution maps, which can increase the ability to identify early and subtle changes^(12)^.

The findings of this study were compared to those of previous studies in pediatric and adult populations^(12-21)^. This data comparison between adults and pediatrics is possible since there are few studies in the pediatric population and because children’s corneal curvature reaches a similar pattern to adults around the age of three ^(22)^. The current study’s population includes children and teenagers aged 8 to 16, allowing for this comparison.

Keratometric parameters (K_flat_, K_steep,_ and K_max_) were similar to those reported in previous studies in pediatric^(12,13)^ and adult populations^(14,15)^ using different tomographic systems (Table 2). When compared to the adult population, our values are lower, which is in accordance with the literature, considering that keratometric values tend to increase with age and go through changes due to genetic and environmental conditions^(23,24)^.

**Table 2:** Keratometric values compared to those from previous studies with different tomographic systems in pediatric and adult populations

		Study with the pediatric population (<18 years)	Study with adult population (≥18 years)		Study with pediatric and adult population
	**Present study**	**Matheus et al.** **- 2017** ^(12)^	**Yağcı et al.** **- 2015** ^(14)^	**Ortiz-Toquero et al.** **- 2016** ^(15)^	**Hashemi et al.** **- 2019** ^(13)^
Equipment	Galilei G6	Pentacam	Galilei	Galilei G4	Pentacam HR
Index	**Mean ± SD**	**Mean ± SD**	**Mean ± SD**	**Mean ± SD**	**Mean ± SD**
K_flat_ (D)	42.37 ± 1.63	42.95 ± 1.32	42.76 ± 1.39	43.23 ± 1.49	42.78 ± 1.11
K_steep_ (D)	43.53 ± 1.65	43.86 ± 1.40	43.71 ± 1.46	44.22 ± 1.38	43.91 ± 1.78
K_max_ (D)	43.90 ± 1.73	44.40 ± 1.45	-	-	-

K_flat_= Flat meridian keratometry; K_steep_= Steep meridian keratometry; K_max_= maximum keratometry.

The CTP, KPI, AAI, coma, asphericity (ε^2^), I-S, BFS radius, and BFS MPE obtained were compared to other studies as shown in Tables 3 and 4, with each study including a description of the equipment used.

The inferior-superior asymmetry ratio, asphericity (ε^2^), and BFS radius were all identical to those observed in prior adult population investigations. The asphericity (Table 3) can aid in the early diagnosis of keratoconus, and Galilei measurements may be reliable, particularly in keratoconic eyes^(25)^. The I-S index (Table 4) has ar numerical range that is similar to that of children (in this study) and adults^(16,17)^, indicating that this is a less parameter throughout life. The BFS radius (Table 4) exhibits the same behavior, but with a more restricted numerical range when comparing children and adult populations, indicating an even more stable parameter^(16)^.

**Table 3:** Tomographic data compared to those of previous studies with a dual Scheimpflug tomographic system in pediatric and adult populations

		Study with pediatric population (<18 years)	Study with adult population (≥18 years)
	**Present study**	**Cakir et al.** **- 2020** ^(18)^	**Smadja et al.** **- 2013** ^(19)^
Equipment	Galilei G6	Galilei G4	Galilei
Index	**Mean ± SD**	**Mean ± SD**	**Mean ± SD**
Eccentricity (ε^2^)	0.28 ± 0.11	-	0.19 ± 0.19
Coma (D)	0.28 ± 0.12	0.25 ± 0.11	-
BFTA MPE (µm)	7.01 ± 2.55	-	8.6 ± 2.8

BFTA MPE= best-fit toric aspheric at its maximum posterior elevation; D= diopter; µm= micrometers.

**Table 4:** Tomographic data compared to those of previous studies with different tomographic systems in pediatric and adult populations

		Study with pediatric population (<18 years)	Study with adult population (≥18 years)			Study with pediatric and adult populations
	**Present study**	**Matheus et al.** **- 2017** ^(12)^	**Gomes et al.** **- 2018** ^(20)^	**Feizi et al.** **- 2016** ^(16)^	**Smadja et al.** **- 2012** ^(17)^	**Wang et al.** **- 2014** ^(21)^
Equipment	Galilei G6	Pentacam	Galilei G6	Galilei	Galilei	Galilei
Index	**Mean ± SD**	**Mean ± SD**	**Mean ± SD**	**Mean ± SD**	**Mean ± SD**	**Mean ± SD**
Corneal thinnest point (µm)	550.20 ± 37.90	547.95 ± 32.06	546.10 ± 23.30	-	550 ± 25	-
KPI (%)	4.03 ± 6.18	-	-	2.32 ± 4.87	-	2.80 ± 4.50
I-S (D)	0.74 ± 0.59	-	-	0.74 ± 0.64	0.58 ± 0.40	-
AAI (µm)	10.44 ± 5.05	-	-	-	16.76 ± 5	-
BFS radius (mm)	6.50 ± 0.32	-	-	6.46 ± 0.18	-	-
BFS MPE (µm)	11.65 ± 4.17	10.54 ± 6.25	-	-	13.10 ± 5.20	-

KPI= keratoconus probability index; I-S= Inferior-superior asymmetry ratio; AAI= Asphericity asymmetry index; BFS radius= Best-fit sphere radius; BFS MPE= Best-fit sphere at its maximum posterior elevation; D: Diopter; µm= micrometers.

There was no difference in the CTP (Table 4) when compared to prior research with pediatric^(12)^ and adult populations^(17,20)^. This parameter is important for differentiating normal corneas from keratoconus ones since it causes progressive thinning ^(17)^. Feng et al. conducted a multicenter study in adult patients, including Brazil, to evaluate the thinnest point of the cornea, and the mean value observed in Brazil was 533 µm, with a variation between 459 (-2SD) and 607 µm (+2SD)^(26)^.

The KPI is considered normal when <10%^(26)^; subclinical keratoconus has usually KPI from 10 to 30% and the keratoconus KPI is superior to 30%^(16,21)^. The present study obtained similar values to adult population studies^(16,21)^ (Table 4).

The AAI <21.5 µm is considered normal^(26)^. Only one of the 68 eyes examined had an AAI of 22 (Table 4). This parameter is an essential screening tool for the keratoconus subclinical form^(17)^. Regarding coma (Table 3), the values found in the present study were similar to values found by Cakir et al.^(18)^.

The elevation indices BFS and BFTA cannot be used interchangeably. BFS MPE values were determined to be within normality and in agreement with the study conducted in an adult population by Smadja et al.^(19)^ (Table 4). The values considered normal were <14 µm, which can help distinguish between corneas with unspecific topographic irregularities and those with subclinical keratoconus.

The BFTA MPE results (Table 4) are primarily utilized to distinguish between the normal cornea, subclinical keratoconus, and keratoconus. Through the BFTA MPE, a cutoff value was established: >13 µm for subclinical keratoconus and >16 µm for keratoconus^(19)^. The present study obtained similar BFTA MPE values to those found in the study by Smadja et al.,^(19)^ and 100% of the analyzed eyes had values within the normal range proposed. This suggests that the same BFTA MPE criteria could be also used in the pediatric population.

Superior corneal asymmetry is defined as the difference >1 D between 90° and 270° in the 3-mm zone^(27)^. Rabinowitz et al.^(28)^ described a superior asymmetry in 16 eyes (4.1%) in a study of 195 normal patients (390 eyes), which may be a typical finding of the topographic examination. Superior asymmetry was identified in 25% of the patients in this study, which contradicts the findings of the cited study. Superior asymmetry in the pediatric population deserves long-term follow-up since there is no consensus on its clinical significance. It is also necessary to prove that there is no structural weakness and that it might progress to inferior corneal asymmetry over time.

CLMI.x is formed by the application of the CLMI strategy (cone location and magnitude of the anterior surface) to not just the anterior axial curvature map, but also the pachymetric map, posterior elevation, and posterior curvature^(29,30)^. According to previous studies, the range value defined for the diagnosis of normality is between 0 and 25, clinical suspicion is between 25 and 80 and keratoconus is between 80 and 100^(29)^. Based on CLMI x, only one eye was considered keratoconus suspect, and one eye was considered keratoconic in the current investigation. Nevertheless, a CLMI.x limitation is that it was originally developed to distinguish keratoconus from normal eyes, and not to identify the subclinical form of keratoconus^(30)^.

This study is one of the rare studies that indicate normal corneal parameters, acquired by a high-precision tomography system in children and adolescents, excluding those with positive allergy skin prick test and specific validated questionnaire. Thus, these parameters may be useful in the early diagnosis of subclinical keratoconus and other corneal ectasias. Nevertheless, more research is needed to evaluate tomographic indexes in a pediatric population with larger sample size, because for variables such as pachy thinnest, a larger sample size is required to get a higher level of confidence and a smaller error.
